# Supplementary material for: Regulation of shoot meristem shape by photoperiodic signaling and phytohormones during floral induction of Arabidopsis
Source: eLife. 2020 Dec 14;9:e60661. doi: 10.7554/eLife.60661 (PMC7771970; doi:10.7554/eLife.60661)
Supplement: Transparent reporting form [file elife-60661-transrepform.pdf]

eLIFE
1st Floor
24 Hills Road
Cambridge CB2 1JP, UK
P 01223 855340
W elifesciences.org
T @elife

eLife's transparent reporting form

We encourage authors to provide detailed information *within their submission* to facilitate the interpretation and replication of experiments. Authors can upload supporting documentation to indicate the use of appropriate reporting guidelines for health-related research (see EQUATOR Network), life science research (see the BioSharing Information Resource), or the ARRIVE guidelines for reporting work involving animal research. Where applicable, authors should refer to any relevant reporting standards documents in this form.

If you have any questions, please consult our Journal Policies and/or contact us: editorial@elifesciences.org.

Sample-size estimation

- You should state whether an appropriate sample size was computed when the study was being designed
- You should state the statistical method of sample size computation and any required assumptions
- If no explicit power analysis was used, you should describe how you decided what sample (replicate) size (number) to use

Please outline where this information can be found within the submission (e.g., sections or figure legends), or explain why this information doesn't apply to your submission:

The appropriate sample-size was not computed.

For the measurement of the shoot apical meristem morphology and cell parameters using MorphoGraphX, 3 to 4 apices were used as described in previous study using this method (Kierzkowski et al., 2012). This information is included in the legends of Figure 1, Figure 3, Figure 7, and Figure 1-Figure supplement 1, Figure 1-Figure supplement 2 and Figure 4-Figure supplement 6.

For the imaging of newly constructed fluorescent reporter lines, at least 3 experiments were conducted where more than 3 meristems were observed. This information is present in the materials and methods section. For the imaging of reported fluorescent reporter lines, at least 4 meristems were observed. This information is included in the legends of Fig. 5, and Fig. 9. We estimated that these observations were suitable to infer the correct localisation of the proteins.
Several replicates of the imaging are presented in the manuscript as described below:
Figure 2: Additional replicates in Figure 2-Figure supplement 2
Figure 4: Additional replicates in Figure 4-Figure supplement 3
Figure 5: Two replicates are present in the figure
Figure 9: Additional replicates in Figure 9-Figure supplement 1

For the RT-qPCR experiments, 3 technical replicates for each of 3 independent biological replicates were used according to "Eleven Golden Rules of Quantitative RT-PCR" (The Plant Cell, Vol. 20: 1736–1737 7, July 2008). This information is included in the materials and methods section.

For flowering-time measurement, more than 10 plants were used for each experiment, as generally accepted by the community. The precise number of plants used for each experiment can be found in the legends of Figure 6, Figure 2-Figure supplement 1, Figure 4-Figure supplement 1, Figure 4-Figure supplement 4 and Figure 6-Figure supplement 1.

Replicates

- You should report how often each experiment was performed
- You should include a definition of biological versus technical replication
- The data obtained should be provided and sufficient information should be provided to indicate the number of independent biological and/or technical replicates
- If you encountered any outliers, you should describe how these were handled
- Criteria for exclusion/inclusion of data should be clearly stated
- High-throughput sequence data should be uploaded before submission, with a private link for reviewers provided (these are available from both GEO and ArrayExpress)

Please outline where this information can be found within the submission (e.g., sections or figure legends), or explain why this information doesn't apply to your submission:

The number of biological replicates is mentioned in the legends in Figure 1, Figure 3, Figure 7, Figure 8, Figure 1-Figure supplement 1, Figure 1-Figure supplement 2, Figure 2-Figure supplement 1, Figure 4-Figure supplement 1, Figure 4-Figure supplement 4 and Figure 6-Figure supplement 1.

We excluded one samples (soc1 19LD replicate I) from the RT-qPCR data of Figure 6A-D and Figure 6-Figure supplement 1C-D (Figure 6-source data 1). No amplification was observed for all genes tested suggesting a problem of RNA quality or during the production of the cDNA synthesis.

Statistical reporting

- Statistical analysis methods should be described and justified
- Raw data should be presented in figures whenever informative to do so (typically when N per group is less than 10)
- For each experiment, you should identify the statistical tests used, exact values of N, definitions of center, methods of multiple test correction, and dispersion and precision measures (e.g., mean, median, SD, SEM, confidence intervals; and, for the major substantive results, a measure of effect size (e.g., Pearson's r, Cohen's d)
- Report exact p-values wherever possible alongside the summary statistics and 95% confidence intervals. These should be reported for all key questions and not only when the p-value is less than 0.05.

Please outline where this information can be found within the submission (e.g., sections or figure legends), or explain why this information doesn't apply to your submission:

We mentioned the sample size and the statistical methods used, in the figure legends and methods section.

(For large datasets, or papers with a very large number of statistical tests, you may upload a single table file with tests, Ns, etc., with reference to sections in the manuscript.)

Group allocation

- Indicate how samples were allocated into experimental groups (in the case of clinical studies, please specify allocation to treatment method); if randomization was used, please also state if restricted randomization was applied
- Indicate if masking was used during group allocation, data collection and/or data analysis

Please outline where this information can be found within the submission (e.g., sections or figure legends), or explain why this information doesn't apply to your submission:

We defined the central zone as the region of 0–2 cells from the central cell at the apex of the shoot, and peripheral zone as the ring 3–5 cells from the central cell. This information is mentioned in the main text and Figure 1-Figure supplement 2A.

Additional data files ("source data")

- We encourage you to upload relevant additional data files, such as numerical data that are represented as a graph in a figure, or as a summary table
- Where provided, these should be in the most useful format, and they can be uploaded as "Source data" files linked to a main figure or table
- Include model definition files including the full list of parameters used
- Include code used for data analysis (e.g., R, MatLab)
- Avoid stating that data files are "available upon request"

Please indicate the figures or tables for which source data files have been provided:

We uploaded 8 additional dataset files associated to Figure 1, 3, 6, 7 and 8 as described below:

Figure 1.
Figure 1-source data 1: Original data of meristem area and cell number of each genotype for Figure 1A-C, E-F and Figure 1- figure supplement 1Dataset 2: Excel file of original data of cell size of each genotype
Figure 1-source data 2: Original data of cell size of each genotype for Figure 1A, D and G and Figure 1- figure supplement 2

Figure 3.
Figure 3-source data 1: Original data of meristem area and cell number of each genotype for Figure 3 A-C and Figure 4-figure supplement 6B-C
Figure 3-source data 2: Original data of meristem area and cell number of each genotype for Figure 3D-F and Figure 4-figure supplement 6D-F

Figure6.
Figure 6-source data 1: Original RT-qPCR data of different genotypes for Figure 6A-D and Figure 6- figure supplement 1
Figure 6-source data 2: Original data of leaf number of different genotypes for Figure 6E-G

Figure 7.
Figure 7-source data 1: Original data of meristem area and cell number of each genotype for Figure 7B-C
Figure 7-source data 2: Original data of cell size of each genotype for Figure 7D

Figure 8.
Figure 8-source data 1: Original ChIP-PCR data for Figure 8
